# Deep Neural Networks for ECG-Based Pulse Detection during Out-of-Hospital Cardiac Arrest

**DOI:** 10.3390/e21030305

**Published:** 2019-03-21

**Authors:** Andoni Elola, Elisabete Aramendi, Unai Irusta, Artzai Picón, Erik Alonso, Pamela Owens, Ahamed Idris

**Affiliations:** 1 Department of Communications Engineering, University of the Basque Country, 48013 Bilbao, Spain; 2 Computer Vision, TECNALIA Research & Innovation, 48160 Derio, Spain; 3 Department of Engineering Systems and Automatics, University of the Basque Country, 48013 Bilbao, Spain; 4 Department of Applied Mathematics, University of the Basque Country, 48013 Bilbao, Spain; 5 Department of Emergency Medicine, University of Texas Southwestern Medical Center, Dallas, TX 75390, USA

**Keywords:** pulse detection, ECG, pulseless electrical activity, out-of-hospital cardiac arrest, convolutional neural network, deep learning, Bayesian optimization

## Abstract

The automatic detection of pulse during out-of-hospital cardiac arrest (OHCA) is necessary for the early recognition of the arrest and the detection of return of spontaneous circulation (end of the arrest). The only signal available in every single defibrillator and valid for the detection of pulse is the electrocardiogram (ECG). In this study we propose two deep neural network (DNN) architectures to detect pulse using short ECG segments (5 s), i.e., to classify the rhythm into pulseless electrical activity (PEA) or pulse-generating rhythm (PR). A total of 3914 5-s ECG segments, 2372 PR and 1542 PEA, were extracted from 279 OHCA episodes. Data were partitioned patient-wise into training (80%) and test (20%) sets. The first DNN architecture was a fully convolutional neural network, and the second architecture added a recurrent layer to learn temporal dependencies. Both DNN architectures were tuned using Bayesian optimization, and the results for the test set were compared to state-of-the art PR/PEA discrimination algorithms based on machine learning and hand crafted features. The PR/PEA classifiers were evaluated in terms of sensitivity (Se) for PR, specificity (Sp) for PEA, and the balanced accuracy (BAC), the average of Se and Sp. The Se/Sp/BAC of the DNN architectures were 94.1%/92.9%/93.5% for the first one, and 95.5%/91.6%/93.5% for the second one. Both architectures improved the performance of state of the art methods by more than 1.5 points in BAC.

## 1. Introduction

Out-of-hospital cardiac arrest (OHCA) remains a major public health problem, with 350,000–700,000 individuals per year affected in Europe and survival rates below 10% [1,2]. Early recognition of OHCA is key for survival [3] as it allows a rapid activation of the emergency system and facilitates bystander cardiopulmonary resuscitation (CPR). Bystanders should apply an automated external defibrillator (AED), designed to be used with minimal training and to guide the rescuer until the arrival of medical personnel [4]. The main goal of OHCA treatment is to achieve return of spontaneous circulation (ROSC), so that post-resuscitation care can be initiated and the patient can be transported to hospital. Early recognition and post-resuscitation care are two key factors for the survival of the patient, and both these factors require the accurate detection of presence/absence of pulse.

Nowadays, healthcare professionals check for pulse by manual palpation of the carotid artery or by looking for signs of life. However, carotid pulse palpation has been proven inaccurate (specificity 55%) and time consuming (median delays of 24 s) for both bystanders and healthcare personnel [5,6,7,8,9]. Consequently, current resuscitation guidelines recommend the assessment of carotid pulse together with looking for signs of life only for experienced people [10]. Checking for signs of life alone has not been proven to be more accurate. In fact, healthcare personnel show difficulties when discriminating between normal (pulse present) and agonal (absence of pulse) breathing [11,12]. More modern approaches use ultrasound to visually assess the mechanical activity of the heart and detect pulse-generating rhythms accurately [13]. Unfortunately, the required equipment is not available during bystander CPR and very rarely for medical personnel in the out-of-hospital setting. Besides, some studies suggest that the use of ultrasound lengthens the duration of chest compression pauses [14,15], decreasing the probability of survival of the patient. Automatic accurate pulse detectors are still needed to assist the rescuer in monitoring the hemodynamic state of the patient [16].

Cardiac arrest rhythms are grouped into the following 4 categories [10]: ventricular fibrillation (VF), ventricular tachycardia (VT), asystole (AS), and pulseless electrical activity (PEA). When ROSC is achieved the patient shows a pulse-generating rhythm (PR). VF and VT need a defibrillation, and a vast number of algorithms have been proposed to detect them [17,18,19,20]. Among non-shockable rhythms, AS is defined as the absence of electrical and mechanical activity of the heart. PEA shows an organized electrical activity of the heart but no clinically palpable pulse, i.e., the mechanical activity is not efficient enough to maintain the consciousness of the patient [21]. AS rhythms can be discriminated using features that are sensitive to amplitude [22], so the most challenging scenario for pulse detection is the discrimination between PR and PEA rhythms. A precise PR/PEA discrimination would allow an earlier recognition of the arrest, and also the identification of ROSC when treating the OHCA patient.

In the last two decades many efforts have been dedicated to automated methods for PR/PEA discrimination based on several non-invasive biomedical signals monitored by defibrillators. The thoracic impedance (TI) shows small fluctuations (≈40 mΩ) with each effective heartbeat [23,24,25], so it has been proposed for pulse detection alone [26,27] or in combination with the ECG [28,29]. However, many commercial defibrillators do not have enough amplitude resolution to detect TI fluctuations produced by effective heartbeats, and the methods have not been proven to be reliable during ventilations because of the TI fluctuations produced by air insufflation [29]. Other signals such as the photoplethysmogram [30,31], capnogram [32], or acceleration [33] have been also included in algorithms for PR/PEA discrimination, but these signals are not commonly available in all monitor/defibrillators. Instead, the ECG acquired using the defibrillation pads is available in all defibrillators, and algorithms based exclusively on the ECG could be of universal use, and easy to integrate in any device.

The main objective of this study was to develop a pulse detection algorithm based exclusively on the ECG acquired by defibrillation pads. Previously a machine learning technique was proposed using a random forest (RF) classifier based on hand-crafted features [34]. Alternatively, deep neural networks (DNN) have shown superior performance in classification problems with large datasets in many fields [35,36,37,38]. DNN solutions have no need of feature engineering as the signals are directly fed to the network which does the exploratory data analysis. Convolutional neural networks (CNN) have been successfully used for heartbeat arrhythmia classification [39,40,41] or the detection of myocardial affections [42,43], and recurrent neural networks (RNN) have been proven accurate for diagnostic applications when time dependencies in the signal are important [44,45]. This work proposes and compares various DNN solutions for PR/PEA classification. The manuscript is organized as follows: Section 2 describes the data used in this study; in Section 3 the proposed DNN solutions are described; classical machine learning based approaches are described in Section 4 and used for comparison; Section 5 describes the optimization process of the models and the evaluation methods applied; and in Section 6 and Section 7 the results are presented and discussed.

## 2. Data Collection

The data of the study were a subset of a large OHCA episode collection gathered by the DFW centre for resuscitation research (UTSW, Dallas). Every episode was recorded using the Philips HeartStart MRx device, which acquires the ECG signal through defibrillation pads with a sampling frequency of 250 Hz and a resolution of 1.03 μV per least significant bit.

There were a total of 1561 episodes of which 1015 contained concurrent ECG and TI signals. The TI signal was necessary to identify ECG intervals free of artefacts due to chest compressions provided to the patient during CPR. Episodes were separated in ROSC and no-ROSC groups based on the instant of ROSC annotated by the clinicians on scene. PEA rhythms were extracted from no-ROSC patients. PR rhythms were extracted after the instant of ROSC for patients who showed sustained ROSC.

ECG segments of 5 s were automatically extracted during intervals without chest compressions. Chest compressions were automatically detected in the TI using the algorithm proposed in [46], or in the compression depth signal of the monitor [47]. Then, organized rhythms (PR or PEA) were automatically identified using an offline version of a commercial shock advise algorithm [17]. Three biomedical engineers reviewed the segments to check they contained visible QRS complexes with a minimum rate of 12 bpm. Every segment was annotated as PEA or PR based on the clinical annotations. Consecutive ECG segments were extracted using a minimum separation between segments of 1 s for PEA and 30 s for PR. PEA is more variable than PR, and occurs during the arrest. During PEA, CPR is given to the patient and intervals without compressions are not frequent. After ROSC is identified (PR segments) chest compressions are interrupted, and long intervals of artefact-free ECG are available. A longer separation between PR segments was considered to increase the variability.

A total of 3914 segments (2372 PR and 1542 PEA) from 279 patients (134 with ROSC and 145 without ROSC) comprised the dataset. Patient-wise training and test sets were created (≈80%/20% of the patients). The training set contained 3038 segments (1871 PR) from 223 patients (105 with ROSC). The test set contained 876 segments (501 PR) from 56 patients (29 with ROSC).

**Figure 1:**
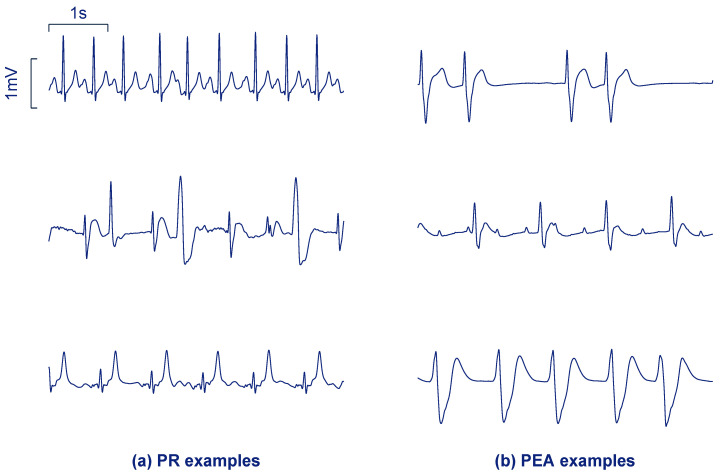
Segments of 5 s corresponding to pulsed rhythm (PR) (**a**) and pulseless electrical activity (PEA) (**b**) from the study dataset.

Figure 1 shows examples of three PR segments (panel a) and three PEA segments (panel b). PR usually shows higher rates, narrower QRS complexes, less heart rate variability and higher frequency content (steeper QRS complexes) than PEA. However, PR in cardiac arrest often shows irregular beats as in the last two examples of Figure 1a. PEA rhythms may show more aberrant QRS complexes, absence of P waves, or more ectopic heartbeats compared to PR.

## 3. Proposed DNN Architectures

Two DNN architectures were implemented for the binary classification of ECG into PR/PEA. The 5 s ECG segments were first bandpass filtered using the typical AED bandwidth (0.5–30 Hz). The filtered ECG was downsampled to 100 Hz to obtain s[n], a signal of N=500 samples, that was fed to the DNN networks. The output of the networks was $p_{PR}\in \left(0,1\right)$, the likelihood that a 5 s segment corresponds to a PR segment. The first solution we propose is a fully convolutional neural network, and the second solution integrates recurrent layers.

### 3.1. First Architecture: Fully Convolutional Neural Network

**Figure 2:**
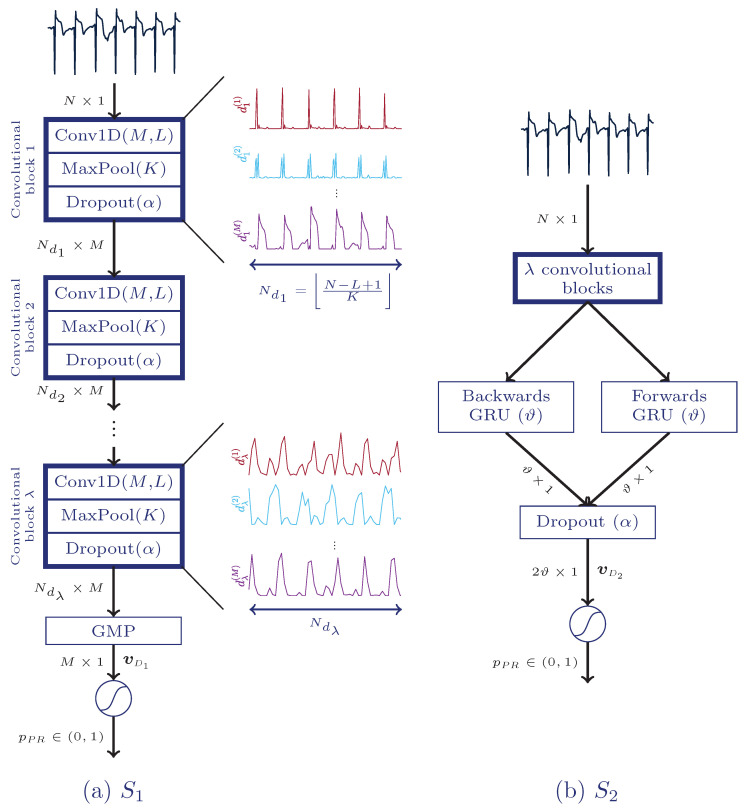
Architectures of the proposed deep neural networks. The fully convolutional solution ($S_{1}$), (**a**), is fed with an electrocardiogram (ECG) segment of *N* samples and includes up to λ convolutional blocks, a global maximum pooling layer (GMP), and a final fully connected layer which provides final likelihood of PR, $p_{PR}$. The $S_{2}$ solution, (**b**), includes up to λ convolutional blocks, a bidirectional gated recurrent unit (BGRU), an extra dropout layer, and a fully connected layer.

Panel a of Figure 2 shows the overall architecture of the first solution ($S_{1}$). It consists of λ convolutional blocks, each one composed of a convolutional, a maximum pooling and a dropout layer.

Convolutional layers apply temporal convolution to the input signal. *M* different convolution kernels of size *L* allows obtaining *M* representations of the signal. The ℓ=1,…,M-th output for the input signal s[n] is calculated as follows:

(1) $$c_{1}^{\left(\ell \right)}\left[n\right]=\phi \left(\sum_{i=0}^{L-1}w_{i}^{\left(\ell \right)}s\left[n-i\right]+b_{i}^{\left(\ell \right)}\right)$$

where *w* and *b* are the weights and biases, respectively, of the convolution kernel (adjusted during training) and ϕ(·) is an activation function. The linear rectifier was adopted as activation function, i.e., ϕ(·)=max{0,·}. Since no padding was applied to s[n] the length of $c_{1}^{\left(\ell \right)}\left[n\right]$ was $N_{c_{1}}=N-L+1$ (the first L−1 samples were discarded). The outputs of the first convolutional layer are fed into a max-pooling layer, which downsamples each input signal by applying the maximum operation with a pool size K=2 to non-overlapping signal segments:

(2) $$p_{1}^{\left(\ell \right)}\left[n\right]=\max{\left\{c_{1}^{\left(\ell \right)}\left[k\right]\right\}}_{k=(n-1)K+1}^{n\cdot K}\;\mathrm{for}\;n=1,\ldots ,\left\lfloor \frac{N_{c_{1}}}{K}\right\rfloor$$

The next step is to apply the dropout operation, which is only present during training and it is a common technique to avoid overfitting. It consists in dropping out some units under a certain probability α at each training step in a mini-batch. When some units are removed different networks are created at each step, so it can be seen as an ensemble technique. Let us denote the outputs of this layer as $d_{1}^{\left(\ell \right)}\left[n\right]$, which will have the same size as $p_{1}^{\left(\ell \right)}\left[n\right]$. Note that once the network is trained $d_{1}^{\left(\ell \right)}\left[n\right]=p_{1}^{\left(\ell \right)}\left[n\right]$.

Pooling layers remove redundant information and reduce the computational cost of the upper layers. The convolution operation of the network permits learning time-invariant features. We added more convolutional blocks with the same number of kernels *M* of size *L*. The outputs of the second convolutional layer are given by the following equation:

(3) $$c_{2}^{\left(\ell \right)}\left[n\right]=\phi \left(\sum_{j=1}^{M}\sum_{i=0}^{L-1}w_{ij}^{\left(\ell \right)}d_{1}^{\left(j\right)}\left[n-i\right]+b_{ij}^{\left(\ell \right)}\right)$$

These outputs are fed into another max-pooling and dropout layers to obtain $d_{2}^{\left(\ell \right)}\left[n\right]$, with *M* different representations of $N_{d_{2}}$ samples. The above equations can be easily adapted to obtain the outputs of the *i*-th convolutional block ($c_{i}^{\left(\ell \right)}$, $p_{i}^{\left(\ell \right)}$ and $d_{i}^{\left(\ell \right)}$) for i=1,…,λ, where λ is the number of convolutional blocks.

The next layer is another pooling layer, namely a global maximum pooling layer. Having *M* different representations of the signal the maximum value of each representation is adopted to obtain a feature vector of *M* elements, i.e.,

(4) $$v_{D_{1}}=\max{\left\{d_{\lambda }^{\left(\ell \right)}\left[n\right]\right\}}_{n}\;\mathrm{for}\;\ell =1,\ldots ,M$$

Finally, a fully connected layer was used as classification stage. This layer is composed of a single neuron with sigmoid activation function to produce $p_{PR}$:

(5) $$p_{PR}=\frac{1}{1+e^{-(w\cdot v_{D_{1}}+b)}}$$

According to [48], it is especially useful to train some layers of the network under the constraint ||w||<γ when using dropout. This additional constraint during the training process reduces overfitting, so every convolutional layer was trained with γ=3.5.

### 3.2. Second Architecture: CNN Combined with a Recurrent Layer

PEA and PR segments show different temporal behaviour. For instance, the time evolution for PR segments is known to be more regular than for PEA segments. These kind of temporal dynamics can be learned by a RNN. So the second solution proposed in this study ($S_{2}$) combines CNN and a bidirectional gated recurrent unit (BGRU), as shown in panel b of Figure 2.

GRU [49] is a simplified version of the well-known long short-term memory (LSTM) [50] with a similar performance [49]. These layers resolve long-term dependencies and avoid vanishing gradient problems. BGRU was inserted between the last convolutional block and the classification stage, removing the global maximum pooling layer. BGRU is composed of two GRU layers, one forward and the other one backward, so more sophisticated temporal features can be extracted by exploiting past and future information at time step *n*. Finally, both outputs were concatenated. A single GRU calculates hidden states $h_{n}$ at time step $n=1,\ldots ,N_{d_{\lambda }}$ based on the past state. Given $D=[d_{\lambda }^{\left(1\right)},\ldots ,d_{\lambda }^{\left(M\right)}]$, the equations of the forwards GRU are described as follows:

(6) $$\begin{array}{ccc} z_{n} & = & \sigma (W_{z}D+U_{z}h_{n-1}+b_{z}) \end{array}$$

(7) $$\begin{array}{ccc} r_{n} & = & \sigma (W_{r}D+U_{r}h_{n-1}+b_{r}) \end{array}$$

(8) $$\begin{array}{ccc} h_{n}^{\prime } & = & \tanh(WD+r_{n}⊙Uh_{n-1}+b) \end{array}$$

(9) $$\begin{array}{ccc} h_{n} & = & z_{n}⊙h_{n-1}+\left(1-z_{n}\right)⊙h_{n}^{\prime } \end{array}$$

where W and U are weight matrices, b is the bias vector, σ(•) stands for sigmoid function, and ⊙ is the Hadamard product. In the equations above $z_{n}$ and $r_{n}$ correspond to the update and reset gates, respectively. The backwards GRU works in the same way but the temporal representations of the input are flipped. The hidden state at the last time step, $h_{n=N_{d_{\lambda }}}$, is fed in to the next layer. Having ϑ units for each direction, a total of 2ϑ features, $v_{D}2=\left[v_{D}2^{\left(1\right)},\ldots ,v_{D}2^{\left(2\vartheta \right)}\right]$, are fed to the last classification layer after applying dropout. The convolutional and recurrent layers were trained under the constraint ||w||<γ.

Another kind of dropout in RNN is recurrent dropout [51], which affects the connections between recurrent units instead of the inputs/outputs of the layer. A recurrent dropout fraction of 0.15 was used to train the final model.

This architecture is optimized simultaneously to obtain the optimal representations of the signal (convolutional layers) and obtain the optimal temporal features (BGRU) for an artificial neural network classifier (fully connected layer).

### 3.3. Training Process

The weights and biases of every layer were optimized using the adaptive moment estimation (ADAM) optimizer [52]. ADAM is a stochastic gradient descent algorithm with adaptive learning rate. According to [52], good default settings are a learning rate of 0.001 and exponential decay rates of 0.9 and 0.999.

The training data were fed into the DNN in batches of 8 during 75 epochs. At the beginning of each epoch training data were shuffled, so the mini-batches at each epoch were different. Additionally, zero-mean Gaussian noise with standard deviation of $10^{-4}$ was added to the signal, and its amplitude was modified by ±2% (uniformly distributed) at each mini-batch. This process enriches the generalization of the model, as the input data for each epoch differs slightly.

The cost function to minimize was the binary cross-entropy:

(10) $$L\left(p\right)=\sum_{i}\eta_{i}\left[y_{i}^{\left(true\right)}\ln\left(p_{PR_{i}}\right)+\left(1-y_{i}^{\left(true\right)}\right)\ln\left(1-p_{PR_{i}}\right)\right]$$

where $y^{\left(true\right)}=\left\{0:\mathrm{PEA},1:\mathrm{PR}\right\}$ are the manual annotations and $\eta_{i}$ are the sample weights. As patients contribute with different number of segments, every patient was weighted equally to train the DNN, so the sum of $\eta_{i}$ within the same patient is equal to 1.

Every experiment was carried out using Keras framework [53] with Tensorflow backend [54]. The DNNs were trained on an NVIDIA GeForce GTX 1080 Ti.

### 3.4. Uncertainty Estimation

The network’s output, $p_{PR}$, represents the likelihood of PR, but it is not an indicator of the prediction confidence of the model. The uncertainty of the DNN decision can be estimated using dropout and data augmentation also during the test phase, a procedure known as Monte–Carlo dropout [55]. For each segment of the test set the prediction is repeated *N* times but adding two random effects: dropout in the DNN network, and the addition of white noise to the ECG. This produces *N* values of $p_{PR}$, and the variance of those values is interpreted as the uncertainty of the prediction. In our experiments *N* was set to 100. The decisions in the test set with an uncertainty above an acceptable threshold were discarded, and in those cases feedback would not be given to the rescuer. The threshold of uncertainty is determined in the training set. The uncertainty of each training instance is computed and the threshold is determined as the uncertainty for which a proportion of feedbacks will be given. In our experiments we tested a proportion of feedbacks from 100% to 80%.

## 4. Baseline Approaches

Machine learning solutions based on well-known ECG features were implemented and compared with $S_{1}$ and $S_{2}$. A total of nine hand-crafted features proposed in [34], $v=[v^{\left(1\right)},\ldots ,v^{\left(9\right)}]$, were computed. They quantify the PR/PEA differences in terms of QRS complex rate and narrowness, slope steepness, spectral energy distribution, and regularity of the signal (fuzzy entropy).

Three classifiers were optimized and trained:

- **RF:** Introduced in [56], RF constructs many weak learners, each trained with a certain proportion of the training data, φ. Each subset is generated by resampling with replacement. Each weak learner is a tree, and only ψ features are considered (drawn randomly from an uniform distribution) at each node. The final decision is made by majority voting. We set the number of trees to 300, and optimized the hyper-parameters φ and ψ.
- **Support vector machine (SVM):** Given a feature vector v, the SVM makes the prediction using the following formula [57]:
  (11) $$y^{\left(pred\right)}=\mathrm{sign}\left(b+\sum_{i=1}^{N_{s}}w_{i}K\left(v,v_{i}\right)\right)$$
  where *b* is the intercept term and $N_{s}$ is the number of support vectors ($w_{i}$ is non-zero only for these vectors). Here K(·,·) denotes the kernel function, which for a Gaussian kernel with $\gamma_{s}$ width is:
  (12) $$K\left(v,v_{i}\right)=\exp(-\gamma_{s}||v-v_{i}{\left|\right|}^{2})$$
  The hyper-parameters soft margin *C* and $\gamma_{s}$ were optimized for the SVM.
- **Kernel logistic regression (KLR):** This is a version of the well-known logistic regression by applying a kernel-trick [57,58]. The prediction is made using Equation (11), and the kernel of Equation (12). The hyper-parameters to optimize were the regularization-term $\lambda_{l}$ and $\gamma_{s}$.

## 5. Evaluation Setup and Optimization Process

### 5.1. Evaluation Setup

The performance of the models was evaluated in terms of sensitivity (Se, probability of correctly identifying PR), specificity (Sp, probability of correctly identifying PEA), and balanced accuracy (BAC, arithmetic mean between Se and Sp). The balanced error rate (BER) was defined as 1−BAC. As patients have different numbers of segments, every patient was weighted equally to compute the performance metrics.

### 5.2. Hyper-Parameter Optimization Process

The hyper-parameters of every model were optimized using Bayesian optimization (BO) [59]. BO is a probabilistic model based approach that attempts to minimize an objective function associated with a real-valued metric, and the variables to optimize can be discrete or continuous. Recent studies report that BO is more efficient than grid search, random search, or manual tuning since it requires less time and the overall performance on the test set is better [60].

BO approximates the objective function to a surrogate function that is cheaper to evaluate. At each iteration a candidate solution is tested to update the surrogate using the past information. With more iterations the approximation of the surrogate is better. BO algorithm variants differ on how this surrogate is constructed. In this study we considered tree-structured parzen estimators (BO-TPE, to optimize $S_{1}$ and $S_{2}$) and Gaussian processes (BO-GP, to optimize RF, SVM, and KLR) [60,61].

The training data were divided patient-wise into 4 folds, and the cross-validated BER was the objective function to minimize. The search space for all models is shown in Table 1.

## 6. Results

**Figure 3:**
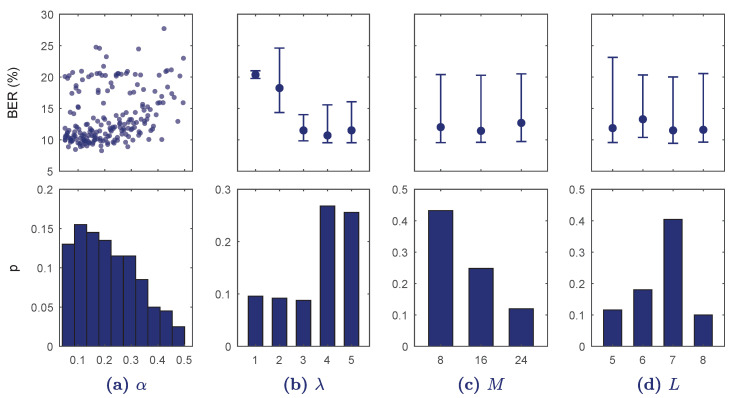
Results of the Bayesian optimization with tree-structured parzen estimators (BO-TPE) optimization algorithm for every hyper-parameter range in $S_{1}$. In the top row balanced error rate (BER) is shown for each continuous value (**a**) or for each discrete value as median and 10–90 percentiles (**b**–**d**). The bottom figures show the probability of selection of the hyper-parameter values in the BO-TPE algorithm.

The results of the BO-TPE algorithm applied for $S_{1}$ are shown in Figure 3. For each hyper-parameter the values of the cross-validated BER are given, continuous for α and median (10–90 percentiles) for the other discrete hyper-parameters. The distributions of the values selected by the optimizing algorithm are also shown (as histogram for α). The number of convolutional blocks, λ, and the dropout rate, α, turned out very determinant. Values of α>0.3 rapidly increased the BER, and including up to λ=4 blocks was the most selected option by the optimization algorithm. The values of *M* and *L* in the selected range had small effect on the performance of the classifier.

**Figure 4:**
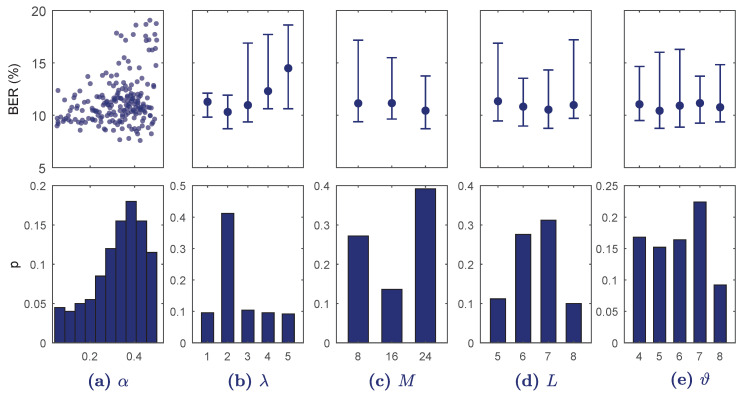
Results of the BO-TPE optimization algorithm for every hyper-parameter range in $S_{2}$. On the top BER is shown for each continuous value (**a**) or for each discrete value as median and 10–90 percentiles (**b**–**e**). The bottom figures show the probability of selection of the hyper-parameter values in the BO-TPE algorithm.

Figure 4 shows the results of BO-TPE for $S_{2}$. Increasing λ overfitted the model rapidly and BER was minimum for λ=2; less convolutional blocks did not provide detailed enough features and increasing λ overfitted the model. Another influential hyper-parameter was α, which showed minimum BER values around 0.4. The hyper-parameters *M*, *L*, and ϑ had little effect on BER.

**Figure 5:**
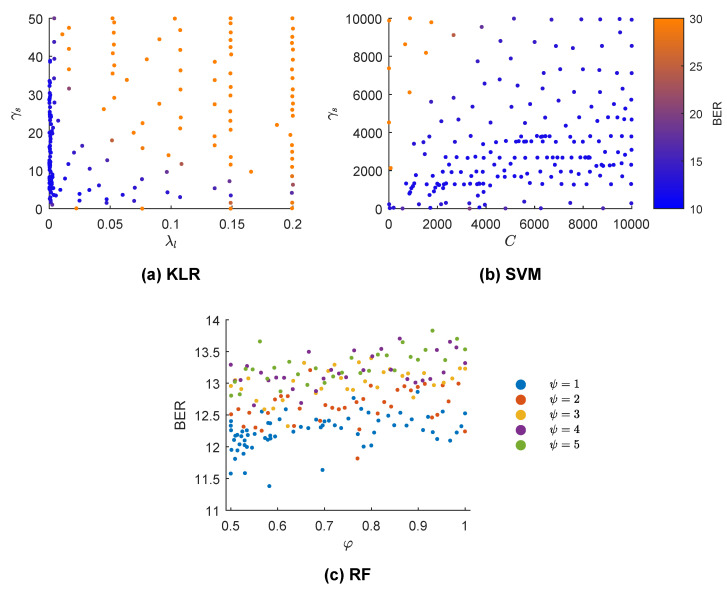
Bayesian optimization with Gaussian processes (BO-GP) results for three different machine learning models. The BER is color-coded in (**a**,**b**) (kernel logistic regression (KLR) and support vector machine (SVM) classifiers) and each point represents the selected solution of the BO-GP in some iteration. In (**c**) (random forest (RF) classifier), discrete values of ψ are color-coded and BER plotted for a range of values for φ.

Figure 5 shows the results of the BO-GP algorithm applied to tune the hyper-parameters of the KLR, SVM, and RF models. The cross-validated BER is color-coded (KLR and SVM) or depicted in the vertical axis (RF). Each point shows a single hyper-parameter combination tested by the BO-GP. In the case of KLR, both hyper-parameters were important, but low values of $\lambda_{l}$ especially yielded lower BER values. For the SVM, low values of *C* and high values of $\gamma_{s}$ produced the worst results, but the selection in the range of values was not as critical as in the KLR solution. Lastly, for RF ψ=1 was the best option, particularly for 0.5<φ<0.6, although the fine tuning of φ was not critical.

Table 2 shows the overall test results of the baseline models and the deep learners in terms of Se, Sp, and BAC, and the set of selected hyper-parameters tuned during the optimization process. There were no differences between the RF, SVM, and KLR models, and any of the deep learning solutions outperformed the baseline models by nearly two percentage points of BAC. Although there was no difference in performance between $S_{1}$ and $S_{2}$, the training process of $S_{1}$ is simpler with less trainable parameters than $S_{2}$ (1441 vs. 4777).

In Table 3 the computation time of the different models is compared. The mean time required to classify the 5 s segment is given, separately for the baseline classifiers in terms of required time for feature extraction ($t_{1}$) and classification ($t_{2}$). Processing times were calculated on a single core of an Intel Xeon 3.6 GHz. As shown in Table 3 the fully convolutional solution, $S_{1}$, was by far the fastest one followed by the baseline models.

**Figure 6:**
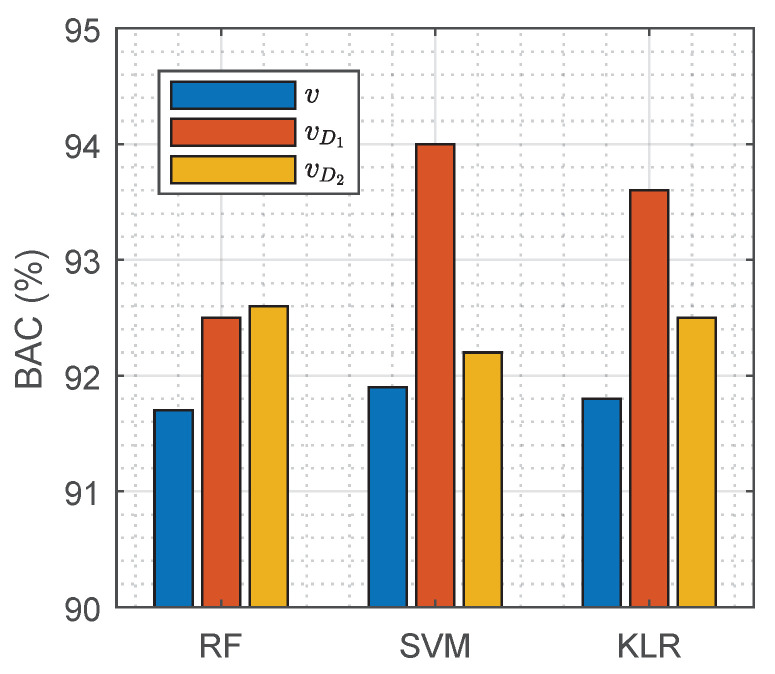
Performance of RF, SVM, and KLR classifiers with hand-crafted features (v), and features extracted by the deep learning architectures $S_{1}$ and $S_{2}$ ($v_{D_{1}}$ and $v_{D_{2}}$ respectively).

Comparative analyses were performed between the 9 hand-crafted features of the baseline models (v) and the features learnt by DNN solutions $S_{1}$ and $S_{2}$ ($v_{D_{1}}$ and $v_{D_{2}}$ respectively). The area under the curve (AUC) for v ranged between 0.88 and 0.94, showing that they had been wisely selected in different domains as described in [34]; but the M=8 features ($v_{D_{1}}$) that $S_{1}$ extracted reported high discriminative values from 0.61 to 0.97, showing that the deep architecture found some very selective features. Next, feature sets from the deep learners $v_{D_{1}}$ and $v_{D_{2}}$ were fed into the baseline classifiers to compare their performance with that of the original v. The BO-GP optimization procedure was repeated for the RF, SVM, and KLR classifiers and results for the test set are depicted in Figure 6. Training the classifiers with $v_{D_{1}}$ and $v_{D_{2}}$ yielded higher BAC values than those obtained with the pre-designed v features. This experiment shows that features defined by the neural networks integrate information not considered by the hand-crafted features, and that they can be successfully used with other classifiers.

**Figure 7:**
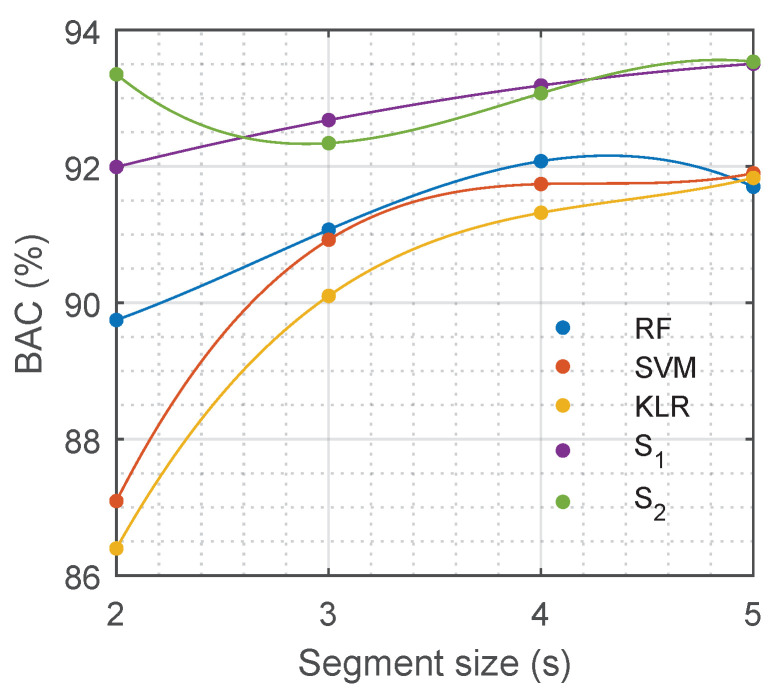
Performance of different models in terms of balanced accuracy (BAC) depending on the duration of the input ECG segment.

The duration of the ECG segment fed into any of the solutions is critical when using a pulse detection algorithm during OHCA treatment. During CPR the ECG signal is strongly affected by chest compression artefacts and electrical defibrillation attempts. For any diagnosis based on the ECG, intervals free of artefact must be used, i.e., extracted either during pauses for rhythm analysis or during chest compression pauses. The segment length used in this study is below the typical interruption for a rhythm analysis, which is between 5.2–26.3 s [62]. However, decreasing the length of the analysis segment would contribute to shorter interruptions in compressions for pulse detection. Reducing hands-off intervals that compromise oxygen delivery to the vital organs increases survival rates [63,64]. Consequently, the solutions of this proposal were tested for different segment durations, from 5 s down to 2 s. The models that were trained for 5-s ECG segments were used, features were extracted using the first seconds of the segment, and those features were fed into the baseline models. The DNN models were fed with the same first seconds of the ECG segments used for feature calculation (note that $S_{1}$ and $S_{2}$ can work with any segment duration at the input). As shown in Figure 7 the best performance for the baseline models was obtained for segment lengths of 5 s. The DNN models outperformed the best baseline models for any segment length, including segments as short as 2 s.

A last evaluation of $S_{1}$ was performed to assess the influence of the degree of uncertainty in the decision of the model. Table 4 shows the performance of the model if the system was designed to give feedback only in a percentage of the analyses, those in which the uncertainty of the decision was lowest. Different percentage thresholds were tested in the training set, from 100% (always give feedback) to 80% (give feedback when the uncertainty is low). Assuming no feedback in 5% of the cases increased the BAC by one percentage point, and the BAC increased up to 97.6% if the system was designed to discard the 20% of the analyses with largest uncertainty.

## 7. Discussion and Conclusions

Pulse detection during OHCA is still an unsolved problem, and there is a need for automatic methods to assist the rescuer (bystander or medical personnel) to decide whether the patient has pulse or not [10]. Non-invasive pulse detection is still a challenging problem [16], and no solutions are currently integrated in monitors/defibrillators. To the best of our knowledge, this is the first study that uses DNN models to discriminate between PR and PEA rhythms using exclusively the ECG.

The two DNN models proposed in this study outperformed the best PR/PEA discriminators based exclusively on the ECG published to date. A RF classifier based on hand-crafted features was proposed in [34] and reported Se/Sp of 88.4%/89.7% for a smaller dataset. A DNN model using a single convolutional layer followed by a recurrent layer was introduced in a conference paper [65], but the Se/Sp/BAC were 91.7%/92.5%/92.1% on the dataset used for this study, that is the BAC was 1.5 percentage points below the current solution. Other DNN solutions were tested in another conference paper [66], where we reported BAC values of 91.2% and 92.6% for preliminary versions of $S_{1}$ and $S_{2}$. Performance was improved in this study adding a general DNN architecture with multiple convolutional layers, a Bayesian optimization procedure which provided insights into the critical hyper-parameters of the networks (see Figure 3 and Figure 4), and a better data augmentation procedure. All these factors contributed to an improved BAC of 93.5% for $S_{1}$ and $S_{2}$, an increase of nearly 2 points from a baseline BAC around 92%, i.e., achieving 20% of the available margin for improvement (8 points) on our initial architectures. Furthermore, we also introduced a new usage framework in which the algorithm was able to automatically assess the uncertainty of the decision, and improved feedback by only reporting decisions with low uncertainties.

There was no difference in terms of BAC between $S_{1}$ and $S_{2}$. The second solution is more complex and should be able to capture more sophisticated features of the signal. However, the number of trainable parameters was 1441 in $S_{1}$ and 4777 in $S_{2}$. Increasing the number of trainable parameters makes the DNN model prone to overfitting, the model “memorizes” the training data loosing generalization capacity and shows poorer performance with unseen data [67,68]. In fact, $S_{2}$ showed higher accuracies during training than $S_{1}$ (98.5% vs. 96.6%). Besides, training was computationally more costly for $S_{2}$, optimizing $S_{1}$ required ≈37 h and optimizing $S_{2}$≈82 h. However, it is possible that with larger datasets $S_{2}$ could generalize better and provide a more accurate model, but OHCA datasets with pulse annotations are costly.

DNN architectures are capable of automatically learning the discrimination features. Our results show that the features learned by $S_{1}$ and $S_{2}$ produced more accurate PR/PEA classifiers than hand-crafted features when fed to the classical machine learning models (see Figure 6). The DNN architectures were able to capture some important ECG characteristics for the identification of pulse that are not accounted for in the hand-crafted features proposed in the literature. In particular, the most discriminative features were those learned by $S_{1}$, which when fed to an SVM classifier boosted the BAC from 92% for hand-crafted features to above 94%.

One of the salient features of the proposed DNN solutions is that they are based solely on the ECG. The ECG is available in all defibrillators/monitors used to treat OHCA patients, so it could be integrated into any equipment. PR/PEA discrimination algorithms that use the ECG and TI have also been proposed [28,29], the TI adds relevant information because effective heartbeats may produce small fluctuations in the TI [23,24]. The BACs of ECG/TI-based PR/PEA discriminators using classical machine learning approaches were around 92% for smaller datasets [28,29]. Defibrillators measure the impedance to check that pads are properly attached to the patient’s chest, that is the reason why the TI signal is not recorded with mΩ amplitude resolution in many devices. In any case, multi-modal deep learning solutions could be explored to increase the accuracy by designing DNN solutions that use both the ECG and TI signals. Moreover, $S_{1}$ extracted significant features, so it could be used as a feature extractor and those features could be combined with features derived from the TI, and other surrogate measures of the hemodynamic state of the patient.

Another critical factor of automatic PR/PEA discrimination algorithms is the ECG segment length needed for an accurate decision. PR/PEA discrimination algorithms need an ECG without chest compression artefacts, this means that compressions have to be interrupted for pulse detection. Pauses in chest compressions compromise the survival of the patient [63,64]. Therefore, current guidelines recommend interruptions of less than 10 s for pulse checks [4,10], but in practice these interruptions are longer than 10 s in more than 50% of cases [14,15]. Our DNN models were very accurate for a segment length of 5 s. Moreover, the length of the segment could be shortened down to 2 s without compromising the BAC of our models (see Figure 7). Consequently, our automatic algorithm could be used to reliably detect pulse during OHCA with interruptions as short as 2–3 seconds, and could be used to avoid the excessively long pauses in chest compressions for pulse detection observed during OHCA treatment.

Measuring the uncertainty of the prediction may be useful when misclassifying an input has a considerable cost, for instance a false pulse indication may unnecessarily interrupt a life saving therapy like CPR. Many efforts have been made to estimate the uncertainty in DNN models, but it is still a challenging problem [69,70,71,72,73]. In this work the uncertainty of the decision was measured using a method known as Monte–Carlo dropout [55], and we found that only giving feedback when the uncertainty was low considerably increased the BAC. For instance, giving a feedback in the 95% of the cases improved the BAC by more than 1 point, and only giving feedback in 80% of cases increased the BAC by over 4 points. During OHCA treatment CPR should be continued until a reliable pulse detection is identified by the algorithm, and the pauses in compressions for the potential feedbacks (reliable or unreliable) will be short, since our algorithms only require ECG segments of 2–3 s. Further work should be done to improve the estimate of the uncertainty of the decision, so that BACs of 97% could be obtained by discarding less than 20% of the potential feedbacks.

In conclusion, this study introduces the use of deep neural networks to discriminate between pulseless and pulsatile rhythms during OHCA using only the ECG. The proposed DNN models outperformed hand-crafted feature-based machine learning solutions, and were able to accurately detect pulse with ECG segments as short as 2–3 s. Moreover, a first attempt at a quantification of the uncertainty of the decision was also introduced to improve the reliability of the feedback given to the rescuer. The proposed solution is based exclusively on the ECG and could be integrated into any monitor/defibrillator.

## Figures and Tables

**Table 1:** Search space of Bayesian optimization (BO) for all models. Here U(min,max) denotes a uniform distribution between min and max values.

Model	Hyper-Parameters
RF	ϑ=U(0.5,1)
	ψ={1,…,9}
SVM	C=U(0.001, 10,000)
	$\gamma_{s}=U$(0.001, 10,000)
KLR	$\lambda_{l}=U\left(0.0001,0.2\right)$
	$\gamma_{s}=U\left(0.0001,15\right)$
$S_{1}$	λ={1,2,3,4,5}
	M={8,16,24}
	L={5,6,7,8}
	α=U(0.05,0.5)
$S_{2}$	λ={1,2,3,4,5}
	M={8,16,24}
	L={5,6,7,8}
	α=U(0.05,0.5)
	ϑ={4,5,6,7,8}

**Table 2:** Summary of the performance of the deep learners and baseline models with the test set and the optimal hyper-parameters chosen by the Bayesian optimization with Gaussian processes (BO-GP) and Bayesian optimization with tree-structured parzen estimators (BO-TPE) algorithms with 5-s electrocardiogram (ECG) segments. DNN models outperformed baseline models in terms of BAC.

	Se (%)	Sp (%)	BAC (%)	Hyper-Parameters
**Baseline models**				
RF	96.0	87.4	91.7	{φ,ψ}={0.58,1}
SVM	97.6	86.2	91.9	$\left\{C,\gamma_{s}\right\}=\left\{2038,1246\right\}$
KLR	97.5	86.2	91.8	$\left\{\lambda_{l},\gamma_{s}\right\}=\left\{0.0013,7\right\}$
**DNN models**				
$S_{1}$	94.1	92.9	**93.5**	{λ,M,L,α}={4,8,7,0.2}
$S_{2}$	95.5	91.6	**93.5**	{λ,M,L,α,ϑ}={2,24,6,0.4,6}

**Table 3:** Computation time to classify a 5-s segment for the baseline and deep neural network (DNN) models. The fastest model was $S_{1}$.

	$t_{1}$ (ms)	$t_{2}$ (ms)	Total (ms)
**Baseline models**			
RF	63.5	0.28	63.8
SVM	63.5	0.35	63.9
KLR	63.5	0.25	63.8
**DNN models**			
$S_{1}$	-	-	**1.6**
$S_{2}$	-	-	101.1

**Table 4:** Performance of $S_{1}$ with different degrees of uncertainty. Scores are given for the test set and the percentage of feedback in the test set are reported. The threshold for feedback was set in the training set.

Training Percentage	Testing Percentage	Se (%)	Sp (%)	BAC (%)
80	78.5	100	95.2	97.6
90	89.6	96.6	93.2	94.9
95	95.4	97.1	92.2	94.6
97.5	98.1	96.3	92.1	94.2
100	100	94.1	92.9	93.5

## Abbreviations

The following abbreviations are used in this manuscript:

ADAM	Adaptive moment estimation
AED	Automated external defibrillator
AS	Asystole
AUC	Area under the curve
BAC	Balanced accuracy
BER	Balanced error rate
BO	Bayesian optimization
BO-GP	Bayesian optimization with Gaussian processes
BO-TPE	Bayesian optimization with tree-structured parzen estimators
CNN	Convolutional neural network
CPR	Cardiopulmonary resuscitation
DNN	Deep neural network
ECG	Electrocardiogram
BGRU	Bidirectional gated recurrent unit
KLR	Kernel logistic regression
OHCA	Out-of-hospital cardiac arrest
PEA	Pulseless electrical activity
PR	Pulsed rhythm
RF	Random forest
RNN	Recurrent neural network
ROSC	Return of Spontaneous Circulation
Se	Sensitivity
Sp	Specificity
SVM	Support vector machine
TI	Thoracic impedance
VF	Ventricular fibrillation
VT	Ventricular tachycardia
